# Psychometric Characteristics of the Oxford Grief Memory Characteristics Scale and Its Relationship With Symptoms of ICD-11 and DSM-5-TR Prolonged Grief Disorder

**DOI:** 10.3389/fpsyt.2022.814171

**Published:** 2022-03-16

**Authors:** Kirsten V. Smith, Jennifer Wild, Anke Ehlers

**Affiliations:** ^1^Department of Experimental Psychology, University of Oxford, Oxford, United Kingdom; ^2^Oxford Health NHS Foundation Trust, Oxford, United Kingdom; ^3^The Loss Foundation [Registered Charity 1147362], London, United Kingdom

**Keywords:** prolonged grief disorder (PGD), bereavement, memory, structural equation modelling (SEM), cross-lagged analyses, cognitive behavioural therapy

## Abstract

Difficulties with loss-related memories are hypothesised to be an important feature of severe and enduring grief reactions according to clinical and theoretical models. However, to date, there are no self-report instruments that capture the different aspects of memory relevant to grieving and adaptation after bereavement over time. The Oxford Grief–Memory characteristics scale (OG-M) was developed using interviews with bereaved individuals and was subject to exploratory and confirmatory factor analyses in a community sample (*N* = 676). Results indicated the scale was unidimensional and demonstrated excellent psychometric properties. The impact of memory characteristics on symptoms of Prolonged Grief Disorder (PGD) according to both ICD-11 and DSM-5-TR criteria were investigated using cross-lagged structural equation modelling in a three-wave longitudinal sample (*N* = 275) at baseline and 6 and 12 months later. Results indicated that loss-related memory characteristics predicted future symptoms of PGD after controlling for autoregressions, and concurrent associations between symptoms and memory characteristics. Cross-lagged associations between memory characteristics and symptoms were significant in the first 6 months of follow-up. After that, memory characteristics predicted future symptoms, but not the other way round. Theoretical and clinical utility of the scale and its features are discussed.

## Introduction

Following a bereavement, severe and enduring grief is thought to persist in approximately 7–14% of grievers (1, 2). In recent years several sets of diagnostic criteria have been developed to measure severe and enduring grief (3–5). While the respective criteria and thresholds for diagnosis differ, all describe persistent yearning or longing for the deceased person and disturbances in thoughts, feelings and behaviours that result in an impairment of functioning.

Theoretical models of prolonged grief hypothesise that symptoms of grief result from a failure to integrate information about the reality of the loss into the autobiographical memory base (6–8). Shear and colleagues proposed that grief symptoms arise as a result of a mismatch between the reality of the death and the mental representation of an attachment figure as being both emotionally and proximally available (8). Boelen and van den Hout (6) similarly suggest that there is a failure to integrate the reality of the loss into the person’s existing mental representation of one’s self and the world. Maccallum and Bryant (7) emphasise the role of mourning to revise the self-memory system, a system proposed to reciprocally link to the autobiographical memory database (9). They propose that symptoms arise as a result of the discrepancy between an individual’s internal self-identity and real-life experiences that challenge the coherence of that identity. Grief adaptation and resolution of symptoms are thought to result from loss-related memory integration within the autobiographical memory base (6, 7) or attachment-related long-term memory (8). Given the important role of memory integration in prolonged grief, an investigation of the characteristics of loss-related memories is of clinical and theoretical interest.

Posttraumatic Stress Disorder (PTSD), like prolonged grief, can also be conceptualised as a disorder that arises from a failure of memory integration of an extremely upsetting event (10, 11). For example, investigations into the characteristics of unwanted intrusive memories in PTSD revealed that these are distressing with vivid perceptual content, disconnected from their context, and are experienced in the “here and now” (12, 13) and thus lead to a sense of current threat for the individual (10). Understanding these characteristics has directly informed advances in therapeutic interventions. For example, stimulus (trigger) discrimination (14) is a therapeutic procedure technique aimed at enhancing the discrimination between triggers in everyday life and trauma memories by training individuals to identify and focus their attention on differences between the trigger in the present moment and its context and the corresponding stimulus during the trauma (10, 15).

Previous investigations of the characteristics of memory associated with severe and enduring grief have focused on the content of intrusive imagery (16) and overgeneral memory (17, 18). Our focus on loss memory characteristics draws on the concept of re-experiencing from the PTSD literature and on in-depth qualitative research investigating memory characteristics in PGD. For example, characteristics of involuntary memories are understood to extend beyond pictures in the mind’s eye to include bodily reactions, sudden emotions that are out of context, “affect without recollection,” see (10), and also behavioural impulses such as running away or an urge to try to find the deceased person. Recent research with bereaved individuals with and without a probable diagnosis of prolonged grief investigated the triggers, valence, content, intrusiveness, unrealness, and physical and emotional consequences of loss-related memories (i.e., memories associated with the deceased or their death) (19). Four themes emerged related to intrusive imagery: memories that indicated a change for the worse, illness-related imagery, positive memories of the lost person and images of the deceased in the present. Further themes in this work described qualities of memory such as negative memories taking precedent over other memories of the deceased, happy memories causing pain, and memories being characteristically distressing, vivid, with a sense of reliving the memory or the presence of the deceased. Participants who experienced prolonged grief were more likely to describe triggers for their loss memories. These were associated with specific times (e.g., time of day or year), seeing other couples or families and internal emotional or physical states.

The content of the interviews informed a series of items aimed to measure multiple features of memory associated with symptoms of PGD. These include descriptions of loss-related memories as intrusive and distressing in people with PGD (19), as well as memories of the deceased being closely connected to the death event (e.g., *“When I try to remember good times we have shared, memories of the loss pop up*”) giving rise to predominantly negative emotions (e.g., *“Even nice memories cause me to feel significant pain*”). Distressing memories were reported as easily triggered (e.g., “*Many different things trigger distressing memories of the loss*”) and activated in response to specific cues such as seeing others with their partners or families. Prior memories of the past and experiences with the deceased highlighted the incongruence of self-image since the loss (e.g., “*When I remember things we did together it feels like I am no longer the same person*”) and memories without the deceased both positive and neutral were less accessible (e.g., “*I struggle to remember positive times without [-]*”). Memories of the loss were also connected to the emotions felt at the time of the loss (e.g., *“When I remember the loss, I feel the same emotions I felt at the time”*) and had a sense of “nowness” (e.g., ‘*To what extent were you reliving your experience from the loss?’*). Memories also involved a sense of visceral changes in the bereaved individual’s body in the form of pain (e.g., “*The physical pain of loss is something I carry everywhere”*), or physical deficit (e.g., “*The loss feels as if part of my body is missing”*).

To date, there is no self-report scale that captures the memory characteristics that may be associated with symptoms of prolonged grief. Such a tool could aid clinicians in their decision-making by highlighting the characteristics of memory associated with prolonged grief that need to be targeted in treatment. The scale could also perform as a process measure allowing investigation of potential mediation effects to better understand clinical outcomes. Finally, the scale could inform adaptations to treatment for individual patients. For example, if a patient endorsed high triggering of intrusive memories by many cues in everyday life, this would indicate clinical intervention focused on stimulus discrimination prior to loss memory work. Previous research has highlighted the importance of exposure to distressing memories in treating prolonged grief (20) but to our knowledge, there are no questionnaires that would give clinicians direction on which aspects of memory associated with severe and enduring grief to focus on or that could be used to monitor the process of changes in loss memories with treatment.

To address this gap, we present a new loss-specific measure, The Oxford Grief Memory Characteristics Scale (OG-M). Developed from interviews described in Smith, Rankin and Ehlers (19), this instrument assesses the characteristics of loss-related memories following bereavement. The aim of this paper is to provide psychometric reliability and validity, including factorial and predictive validity for the OG-M and the OG-M-S, a shortened clinician version for use in clinical settings. Furthermore, we aimed to establish whether the memory characteristics measured with the OG-M predict higher grief severity over time when accounting for prior symptom levels.

## Materials and Methods

### Participants and Procedure

Three separate samples of bereaved individuals were recruited via social media advertisements, bereavement charity mailing lists, and the Google content network in the United Kingdom. Demographics and loss characteristics for each sample are described in Table 1. Factorial and psychometric validity and scale reduction were confirmed using 676 adults. Test-retest reliability was assessed with another sample of 50 individuals who completed the OG-M twice with a 1-week gap.

**TABLE 1:** Sample demographics and loss characteristics.

Variable	Sample		
	Cross-sectional	Test-retest	Longitudinal
	(*N* = 676)	(*N* = 50)	(*N* = 275)
Age M (SD)	49.22 (12.52)	51.46 (14.54)	46.43 (13.24)
Women (%)	81.5	84.0	78.5
Months since loss M (SD)	56.91 (79.79)	23.74 (48.44)	2.94 (2.01)
Violent loss (%)	19.5	26.0	9.1
**Who died? (%)**			
Partner	36.1	28.0	30.2
Child	21.0	22.0	8.7
Sibling	6.5	0.0	5.8
Parent	28.3	42.0	38.2
Another close relative or non-relative	8.2	8.0	17.1

*Violent loss defined as resulting from human (in)action (i.e., suicide, homicide, accident, unintentional overdose, and medical negligence) vs. illness.*

A third three-wave longitudinal sample assessed in the first 6 months after bereavement and again 6 and 12 months later consisted of 275 adults and was used to assess the effect of the scale over time.

Questionnaire data were collected online and followed an ethical framework for internet research (21). All participants were compensated for their time and informed consent was obtained electronically in accordance with ethical approval given by the University of Oxford Medical Sciences Inter-Divisional Research Ethics Committee (MS-IDREC-C1-2015-230; MS-IDREC-C1-2015-231).

### Measures

#### Cognitive Measures

##### The Oxford Grief–Loss-Related Memory Characteristics Scale (OG-M)

Questionnaire items were developed from interviews with bereaved individuals to assess difficulties with memory following their loss (19). Face and content validity were determined in collaboration with bereaved service users and therapists experienced in the treatment of traumatic bereavement leaving 27 questionnaire items from a potential pool of 40 items. The questionnaire asks participants to rate on a 5-point scale (0–not at all to 4–very strongly) the extent to which each statement regarding their memory of the loss applied to them during the last month. Twenty-three items probed memory triggers and their consequences (e.g., “*I am reminded of the loss for no apparent reason*”), qualities of memory (e.g., “*Memories of things we did together are painful*”), the poor availability of positive memories (e.g., “*I struggle to remember positive times without [-]*”), and the physical impact of loss-related memories (e.g., “*The memories of [-]’s death make my body ache with overwhelming fatigue*”). Four further items, taken from the Intrusions Questionnaire (13), asked about unintentional memories of the loss (frequency in the last week, distress, how much they seemed to be happening now instead of in the past, and the extent to which they felt as though they were reliving the memory).

#### Symptom Measures

##### Prolonged Grief Disorder

At the time this study was conceptualised no criteria for prolonged grief had officially been adopted by ICD-11 or DSM-5-TR. Therefore, data were collected using the 16 symptoms of persistent complex bereavement disorder (PCBD) (5). An extended version of the Prolonged Grief Disorder Scale (PG-13; Prigerson and Maciejewski (22) was used to assess the prevalence and severity of PCBD symptoms. Ten of the symptoms of the PGD criteria presented by Prigerson and colleagues (23) overlap with the symptoms of PCBD (e.g., yearning for the deceased, feelings of emotional numbness/detachment from others, feeling that a part of oneself died along with the deceased). Six additional items were added to the PG-13 items that correspond to the symptoms of the PCBD criteria not represented by the PGD-2009 criteria. Items were measured on a five-point scale with separation distress items rated (0–not at all to 4–at least once a day) and cognitive, emotional, and behavioural grief symptom items rated (0–not at all to 4–overwhelmingly).

The 10 symptom DSM-5-TR criteria can be fully represented with these items (3). For the ICD-11 criteria symptoms (4) we included item 14 from the PCL-5 “trouble experiencing positive feelings” rescaled to match the PG-13 items. All other criteria were covered by PCBD items with the exception of the intense emotional pain of blame symptom. In previous research this has been represented by items pertaining to self-blame (e.g., feelings of guilt) (24–26) therefore blame and guilt were represented by a single item (i.e., feeling bad about oneself because of things that happened in relation to the death or the relationship) (PGD ICD-11, cross-sectional α = 0.90, test-retest α = 0.89, longitudinal α = 0.89; PGD DSM-5-TR-11, cross-sectional α = 0.90, test-retest α = 0.89, longitudinal α = 0.90).

###### Posttraumatic Stress Disorder Checklist for DSM-5 (PCL-5) (27)

The PCL-5 is a self-report instrument assessing distress associated with the 20 symptoms of PTSD in DSM-5 over the past month. Items were rated on a five-point scale, from (0–not at all to 4–extremely). Internal consistency was excellent in all samples (cross-sectional α = 0.94, test-retest α = 0.94, longitudinal α = 0.94).

###### Patient Health Questionnaire (PHQ-9) (28)

The PHQ-9 is a self-report measure that mirrors the nine major depressive symptoms for major depressive disorder according to the Diagnostic and Statistical Manual, 4th Edition, Text Revision [DSM IV-TR (29)]. Each item is scored (0–not at all to 3–nearly every day) in the last two weeks. Internal consistency was excellent in all samples (cross-sectional α = 0.92, test-retest α = 0.92, longitudinal α = 0.91).

###### The Oxford Grief Coping Strategies Scale—Proximity Seeking Subscale

This 23-item questionnaire asks participants on a 5-point scale (1–never to 5–always) to indicate how often they used particular strategies to cope with their loss. Items pertain to four content factors: Avoidance, Proximity seeking, Grief rumination, and Injustice rumination. The 7-item proximity seeking subscale measures the extent to which bereaved individuals engage in behaviours [e.g., *“I feel compelled to touch things that they touched (e.g., belongings, chairs, beds)*”], activities (e.g., “*I neglect other things because I spend a lot of time doing things for them [e.g., creating memorials, fundraising)*”] and experiences (e.g., “*I dwell on the things we won’t get to do together*”) aimed at restoring or maintaining proximity to the deceased person. Composite reliability was good in the cross-sectional sample (ω = 0.87).

### Statistical Analyses

#### Factorial Validity

Factor analyses were conducted using Mplus Version 8 (30). Cross-validation of the factorial model was conducted using a 50% random split of the first dataset (31). The initial measurement model was built using exploratory factor analysis (EFA) on one half of the data and then tested on the other half using confirmatory factor analysis (CFA). The estimation method employed in factor analysis is determined by the distribution of the variables in question. Recent advances in statistical software have meant that variables measured on different scales can be entered into the same model within an EFA and CFA framework using weighted least squares mean and variance adjusted (WLSMV) estimation (30). The OG-M comprises items rated on a 0 (not at all) to 4 (very strongly) with four items taken from the Intrusions Questionnaire (13) originally rated on a different scale, therefore, a WLSMV estimation was employed to handle items with mixed distributions. Geomin oblique rotation was used as scale factors were expected to correlate (30). Conceptual interpretability, eigenvalues greater than 1, a scree plot derived using parallel analysis (32, 33), and model fit statistics were used to determine model adequacy. A χ^2^ goodness-of-fit test where the χ^2^:df ratio is smaller than 3:1 was considered acceptable. A comparative fit index (CFI) or Tucker Lewis Index (TLI) of 0.90 or higher or 0.95 or higher was considered acceptable and good respectively. For root mean square error of approximation (RMSEA) 0.08 or lower was considered acceptable and 0.06 or lower was considered good (34–36). Decisions about factor determinacy were made based on (1) factor loadings greater than 0.35 and (2) items with comparable cross-loadings were ultimately placed on the factor where they loaded most strongly (37). Modification indices were only considered when large (>10) and in line with the conceptual interpretation (37).

#### Psychometric Validation

Composite reliability was assessed by McDonalds’ Omega (ω = (Σ| λi |)^2^/([Σ| λi |]^2^ + Σδii), where λi are the factor loadings and δii, the error variances) for WLSMV on the total scale in the CFA (38). Criterion and convergent validity were determined using correlations with measures of psychopathology (i.e., PGD, PTSD, and depression) and behaviours of proximity seeking to the deceased [i.e., Oxford Grief Coping Strategies Scale (OG-CS)–Proximity Seeking subscale]. The stability of the total scale and subscales over time was measured using the test-retest reliability sample. A correlation greater than 0.70 between two time points a week apart was used to indicate acceptable retest reliability. The average variance extracted (AVE) score was calculated to determine the average variance in the latent factor that is accounted for by its items (39). A score of 0.50 or higher confirms factorial convergent validity (40).

#### Scale Reduction

Next, we aimed to create a clinician-version consisting of a shortened list of items from the original memory characteristics scale that balanced predictive validity with ease of use. The OG-M items were subject to Area Under the Curve (AUC) analysis of Receiver Operating Characteristics curves (ROC) (41). Each item was analysed for its ability to predict a diagnosis of PGD using the DSM-5-TR diagnostic criteria and ICD-11 criteria (3–5). Participants were considered as meeting criteria for ICD-11 PGD if their loss had occurred at least 6 months previously (12 months for DSM-5-TR) and they endorsed at least one item of separation distress daily, and at least 1 of 10 (3 of 8 for DSM-5-TR) symptoms of cognitive, emotional, and behavioural symptoms, resulting in significant impairment of functioning. The AUC indicates the probability that a participant selected at random with a diagnosis will score higher on the OG—M than a participant without a diagnosis (42). A value of above 0.90 is considered an excellent predictor of the outcome, with values above 0.80 reflecting good, and above 0.70 fair (43, 44).

#### Structural Equation Modelling

**FIGURE 1:**
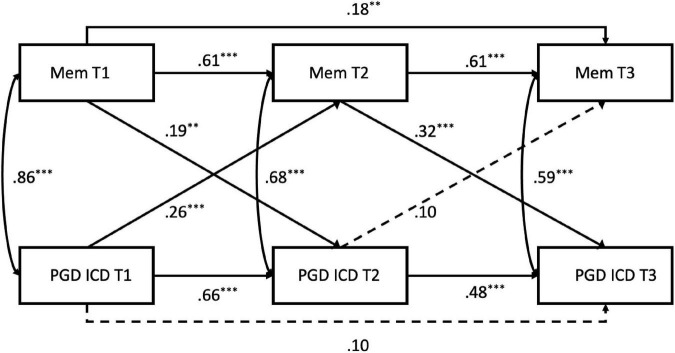
Autoregressive cross-lagged model of PGD ICD-11 and loss-related memory characteristics. Standardised coefficients are shown. Broken lines indicate non-significant paths. MEM, The Oxford Grief Memory Characteristics Scale (OG-M); PGD ICD, Prolonged Grief Disorder according to the ICD-11 diagnostic criteria. Asterisks indicate significant associations (^**^p < 0.01, ^***^p < 0.001). χ^2^ = 1.47, *df* = 2, *p* > 0.05, RMSEA = 0.00, CFI = 1.00, TLI = 1.00.

**FIGURE 2:**
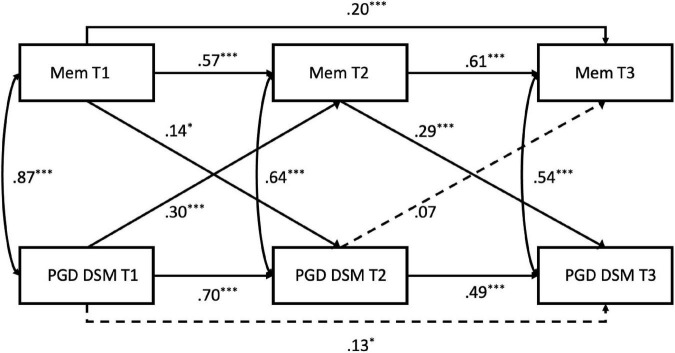
Autoregressive cross-lagged model of PGD DSM5-TR and loss-related memory characteristics. Standardised coefficients are shown. Broken lines indicate non-significant paths. MEM, The Oxford Grief Memory Characteristics Scale (OG-M); PGD DSM, Prolonged Grief Disorder according to the DSM-5-TR diagnostic criteria. Asterisks indicate significant associations (^**^p < 0.01, ^***^p < 0.001). χ^2^ = 5.12, *df* = 2, *p* > 0.05, RMSEA = 0.075, CFI = 1.00 TLI = 0.98.

Finally, in order to determine the predictive role of memory characteristics on grief severity we employed a second-order autoregressive cross-lagged panel model in Mplus Version 8 (30). We present the results for both ICD-11 and DSM-5-TR criteria for PGD. Sum scores for the OG-M and PGD criteria at baseline (0–6 months), short-term follow up (6–12 months) and long-term follow-up (12–18 months) were calculated and autoregressive paths were modelled to account for the influence of variables at preceding time points. Correlated errors between memory characteristics and PGD symptoms at concurrent time points were also added to account for their joint fluctuation over time. Cross lagged paths estimated the extent to which memory characteristics predicted PGD symptoms at a later time point and vice versa after controlling for autoregressive paths and concurrent associations between symptoms and memory characteristics (see Figures 1, 2).

A full information maximum likelihood (FIML) approach implemented in Mplus was used to estimate missing data to minimise the bias associated with attrition. A majority of participants completed the OG-M at all three time points (66.2%) with a high proportion answering at least two (87.6%); no participants were excluded. Covariance coverage, which measures the impact of missing data, ranged from 0.66 to 0.93 for each pair of variables, well above the minimum threshold of 0.10 for model convergence (45). The following fit indices were used to determine adequate fit: χ^2^ *p* > 0.05, CFI > 0.90, TLI > 0.90, RMSEA < 0.01 (34–36).

## Results

### Exploratory Factor Analyses—Loss-Related Memory Characteristics

All 27 items were entered into exploratory factor analyses using WLSMV estimation, which is recommended for outcomes with mixed distributions. Inspecting eigenvalues greater than 1 suggested a three-factor structure. The first factor accounted for a large proportion of the variance with an eigenvalue of 14.12, the two smaller factors had eigenvalues of 1.40 and 1.33, respectively. The scree plot suggested a two-factor solution. The one-factor model indicated a good fit to the data (CFI = 0.99, TLI = 0.98, RMSEA = 0.057 (0.05–0.06), χ^2^ = 636.55 on *df* = 324, χ^2^:*df* = 1.97). The two-factor solution fit statistics indicated a slightly worse fit to the data (CFI = 0.98, TLI = 0.98, RMSEA = 0.065 (0.06–0.07), χ^2^ = 664.72 on *df* = 298, χ^2^:*df* = 2.23) and the three-factor solution, while demonstrating the best fit (CFI = 0.99, TLI = 0.99, RMSEA = 0.051 (0.04–0.06), χ^2^ = 480.51 on *df* = 273, χ^2^:*df* = 1.76), was not conceptually interpretable. No items loaded strongly on the second factor and only one item loaded strongly on the third. The four-factor solution did not converge. Therefore, a one-factor solution was deemed to be the most appropriate fit for the data. Inspection of the modification indices suggested that a correlated error should be added between “*When I remember things we did together it feels like I am no longer the same person*” and “*When I remember something I did in the past; it feels like I am no longer the same person*” (*MI* = 47.89). These correlated errors are likely due to similar wording and as such it seems likely that some of the shared variance between these items can be attributed to item wording instead of the factor on which these items load (46).

### Confirmatory Factor Analyses—Loss-Related Memory Characteristics

The CFA assessed the fit of the chosen one-factor solution with one correlated error using the CFA sample (*N* = 328). The fit statistics for the one-factor indicated a good fit CFI = 0.98 and TLI = 0.98, and a close to good fit for RMSEA = 0.062 (0.06–0.07), χ^2^ = 678.35 on *df* = 323, χ^2^:*df* = 2.10) supporting the chosen solution in the EFA. Table 2 summarises the standardised factor loadings for the one-factor solution using EFA and CFA.

**TABLE 2:** Analyses of the Oxford Grief loss-related memory characteristics scale (OG-M).

		Factor	
		1	
Loss-related memories items		EFA	CFA
1	In the last week, approximately how often did unwanted memories of the loss pop into your mind?	0.70	0.65
2	How distressing were these memories?	0.84	0.81
3	To what extent did they seem to be happening now instead of being something from the past?	0.73	0.78
4	To what extent were you reliving your experience from the loss?	0.87	0.86
5	The memories of [-]’s death make my body ache with overwhelming fatigue.	0.80	0.81
6	The physical pain of loss is something I carry everywhere.	0.85	0.84
7	When I remember the loss it feels unreal.	0.72	0.58
8	Seeing other people with their partners or families makes me painfully aware of my loss.	0.68	0.65
9	Many different things trigger distressing memories of the loss.	0.84	0.83
10	If my mind is distracted from my grief for a while it will hit me like a wave later.	0.81	0.80
11	I am reminded of the loss for no apparent reason.	0.77	0.69
12	When I remember the loss, I feel the same emotions I felt at the time.	0.64	0.70
13	When I try to remember good times we have shared, memories of the loss pop up.	0.69	0.74
14	The loss feels as if part of my body is missing.	0.80	0.80
15	Many things in everyday life trigger overwhelming sadness.	0.83	0.80
16	When I think of [-] all I can remember is their suffering.	0.57	0.54
17	When I think about [-] I will always think about how they died.	0.62	0.60
18	My memories of [-] are so vivid it feels like they are here.	0.47	0.43
19	When I remember things we did together it feels like I am no longer the same person.	0.82	0.67
20	Memories of things we did together are painful.	0.72	0.67
21	Even nice memories cause me to feel significant pain.	0.84	0.77
22	I struggle to remember positive times without [-].	0.66	0.64
23	Looking at a calendar mainly reminds me of the bad things that happened on those days.	0.58	0.68
24	When I remember something I did in the past; it feels like I am no longer the same person.	0.79	0.65
25	I hardly remember anything that I did without [-].	0.69	0.55
26	I feel a strong urge to comfort [-].	0.60	0.67
27	I find myself suddenly overcome to find [-].	0.66	0.67

*EFA (N = 348) CFA (N = 328). Factor labelled as Loss-Related Memory Characteristics. All factor loadings significant to p < 0.05.*

### Psychometric Validation—Loss-Related Memory Characteristics

The total loss-related memories scale demonstrated excellent composite reliability (ω = 0.97) and sufficient convergent validity (AVE > 0.5). Test-retest reliability over 7 days for the OG-M was excellent (*r* = 0.90, *p* < 0.001). Correlations between the total score of the OG-M and symptom measures of PGD (ICD; *r* = 0.84, *p* < 0.001, DSM; *r* = 0.82, *p* < 0.001), PTSD (*r* = 0.77, *p* < 0.001), depression (*r* = 0.65, *p* < 0.001), and behavioural proximity seeking (*r* = 0.70, *p* < 0.001) were all strong and significant, confirming criterion validity. Table 3 summarises the psychometric validation of the OG-M.

**TABLE 3:** Psychometric validity of the OG-Loss-related memory characteristics scale.

Reliability/Validity	Measure	Total scale
Composite	McDonald’s Omega	0.96
Criterion	PGD ICD *r*	0.84***
	PGD DSM	82***
	PTSD *r*	0.77***
	Depression *r*	0.65***
	Proximity seeking (OG-CS) *r*	0.70***
Test-retest	*r*	0.90***
Convergent	AVE	0.50

Test-retest reliability confirmed if r > 0.70. Convergent validity of factors confirmed if AVE > 0.5. r, correlation; AVE, Average variance extracted. ***p < 0.001.

### The Oxford Grief–Memory Characteristics–Short Scale

Balancing overlapping content and AUC statistics calculated on the larger cross-sectional sample 1 (*N* = 676), 11 items were removed from the OG-M to create the Oxford Grief–Memory Characteristics–Short Scale (OG-M-S) (Table 4). Two of the removed items (“The memories of [-]’s death make my body ache with overwhelming fatigue” and “When I remember things we did together it feels like I am no longer the same person”) had AUC values higher than some of the retained items. However, these two items were deemed to overlap in content (i.e., visceral experiences and memories triggering identity disruption) with items scoring higher on AUC and were, therefore, removed for parsimony. The total OG-M-S had an AUC of 0.85 (*SE* = 0.02, *p* < 0.001, *CI* = 0.82–0.88) for PGD ICD-11 and 0.80 (*SE* = 0.02, *p* < 0.001, *CI* = 0.77–0.84) for PGD DSM-5-TR which was comparable with the full OG-M ICD-11; AUC = 0.84 (*SE* = 0.02, *CI* = 0.81–0.87); DSM; AUC = 0.80 (*SE* = 0.02, *CI* = 0.76–0.83). Converting the AUC to Cohen’s *d* using the formulas described in Ruscio (47) gives a very large effect size for both the full and the short OG-M scale (ICD-11; *d* = 1.47, DSM-5-TR *d* = 1.19) in the prediction of PGD (48).

#### Psychometric and Factorial Validity of the Oxford Grief–Memory Characteristics–Short Scale

Exploratory factor analysis of the shortened scale revealed one eigenvalue above 1 (9.09). The one factor solution was a good fit to the data χ^2^ = 225.87 on *df* = 104, χ^2^:*df* = 2.17, CFI = 0.99, TLI = 0.99, RMSEA = 0.059 (0.05–0.07). The two-factor solution had a marginally better fit χ^2^ = 179.45 on *df* = 89, χ^2^:*df* = 2.01, CFI = 0.99, TLI = 0.99, RMSEA = 0.055 (0.03–0.06), but was not optimal because only two items loaded strongly on the first factor. Confirmatory factor analysis of the one factor solution resulted in a good fit CFI = 0.99, TLI = 0.99, χ^2^ = 200.32 on *df* = 104, χ^2^:*df* = 1.92, RMSEA = 0.055 (0.04--0.07)^1^.

The shortened scale had excellent composite reliability ω = 0.97 and good test-re-test reliability *r* = 0.87. Criterion validity correlations with the symptom scales were all strong and significant (PGD: ICD, *r* = 0.84; DSM, *r* = 0.84; PTSD, *r* = 0.76; depression, *r* = 0.65; proximity seeking, *r* = 0.69). The scale also met the requirements of convergent validity AVE = 0.67.

### Cross-Lagged Models of the Oxford Grief–Memory Characteristics Scale

Both the PGD ICD-11 and the PGD DSM-5-T5 cross-lagged models were an excellent fit to the data (ICD, χ^2^ = 1.47, *df* = 2, *p* > 0.05, RMSEA = 0.00 (0.00–0.11), CFI = 1.00, TLI = 1.00; DSM, χ^2^ = 5.12, *df* = 2, *p* > 0.05, RMSEA = 0.075 (0.00–0.16), CFI = 1.00 TLI = 0.98). Parameter estimates are presented for PGD ICD-11 in Figure 1 and PGD DSM-5-TR in Figure 2. For both PGD conceptualisations, memory characteristics predicted PGD symptoms 6 months later after controlling for autoregressions and correlated errors between concurrent symptom and memory characteristics. This effect can be observed from baseline (0--6 months) to short-term follow-up (6--12 months), as well as from short-term to long-term follow-up (12--18 months). In contrast, while PGD symptoms significantly predicted future memory characteristics between baseline and short-term follow-up, this effect was not evident during the long-term follow-up. The results of the memory characteristics short scale (OG-M-S) did not differ from the full OG-M^2^.

**TABLE 4:** Area under the curve analysis of the OG-M items.

Loss-related memory characteristics items—Shortened scale and removed items		ICD AUC	DSM AUC
1	In the last week, approximately how often did unwanted memories of the loss pop into your mind?	0.77	0.75
2	How distressing were these memories?	0.76	0.74
3	To what extent were you reliving your experience of the loss?	0.77	0.73
4	The physical pain of loss is something I carry everywhere.	0.79	0.75
5	When I remember the loss it feels unreal.	0.70	0.67
6	Seeing other people with their partners or families makes me painfully aware of my loss.	0.70	0.67
7	Many different things trigger distressing memories of the loss.	0.76	0.71
8	If my mind is distracted from my grief for a while it will hit me like a wave later.	0.78	0.72
9	When I remember the loss, I feel the same emotions I felt at the time.	0.70	0.67
10	When I try to remember good times we have shared, memories of the loss pop up.	0.72	0.67
11	The loss feels as if part of my body is missing.	0.80	0.77
12	Many things in everyday life trigger overwhelming sadness.	0.78	0.74
13	Even nice memories cause me to feel significant pain.	0.75	0.71
14	When I remember something I did in the past; it feels like I am no longer the same person.	0.77	0.77
15	I feel a strong urge to comfort [-].	0.70	0.67
16	I struggle to remember positive times without [-].	0.73	0.69
R	When I think about [-] I will always think about how they died.	0.69	0.67
R	To what extent did they seem to be happening now instead of being something from the past?	0.70	0.65
R	The memories of [-]’s death make my body ache with overwhelming fatigue.	0.78	0.72
R	I am reminded of the loss for no apparent reason.	0.73	0.68
R	When I think of [-] all I can remember is their suffering.	0.65	0.62
R	My memories of [-] are so vivid it feels like they are here.	0.62	0.60
R	When I remember things we did together it feels like I am no longer the same person.	0.77	0.75
R	Memories of things we did together are painful.	0.73	0.69
R	Looking at a calendar mainly reminds me of the bad things that happened on those days.	0.67	0.67
R	I hardly remember anything that I did without [-].	0.71	0.69
R	I find myself suddenly overcome to find [-].	0.67	0.65

*AUC, Area Under the Curve; R, Removed item.*

## Discussion

The aim of this study was to test the psychometric and factorial properties of the Oxford Grief–Memory Characteristics scales (OG-M and OG-M-S). It was designed to measure the content, triggers, qualities, and consequences of loss-related memories and was developed from interviews with bereaved individuals with and without a diagnosis of PGD (19). Exploratory and confirmatory factor analyses supported a unidimensional solution. Psychometrics indicated the scale had excellent composite and test-retest reliability, criterion validity, and adequate convergent validity. A shortened version of the OG-M, suitable for use in clinical settings, demonstrated good to excellent psychometric and factorial validity. Longitudinal cross-lagged analyses of the memory characteristics scale revealed it as a significant predictor of future symptoms of PGD for both ICD-11 and DSM-5-TR criteria after controlling for the effect of prior symptoms levels and concurrent associations. These results are supported by a previous finding that the OG-M was able to distinguish between trajectories of high and low grief over time (49).

An important finding was that while early memory characteristics and PGD symptoms (0–6 months) showed significant cross-lagged relationships, each measure at baseline predicting the other 6 months later (short-term follow-up), only memory characteristics at 6–12 months predicted the long-term maintenance of PGD symptoms at 12–18 months, whereas symptom severity at 6–12 months no longer predicted memory characteristics at long-term follow-up. This pattern of results would be expected by psychological models of PGD (6, 8) that aim to explain the persistence of grief symptoms beyond the initial months of grieving, where most people will have frequent memories of the death and the deceased together with feelings of loss and grief. People for whom grief does not resolve naturally are thought to differ from those who adapt to their loss by several factors that maintain their symptoms, including memory characteristics, appraisals, social disconnection and unhelpful coping strategies (49–53, 67).

Factor analyses suggested that the memory characteristics measured with the OG-M are explained by one underlying dimension. This could be partly because the sample included a wide range of bereaved people with a wide range in severity of their grief reactions and endorsement of the memory items. It is possible that in a clinical sample of patients with PGD, factor analyses would reveal a more complex factor structure. However, it is also possible that a unidimensional factor structure would be retained as the items of the OG-M can be interpreted as representing different aspects of memory “re-experiencing” driven by poor loss memory integration.

Previous work has suggested that traumatic memories, including traumatic loss memories, that are poorly integrated with other information in autobiographical memory are easily triggered due to poor inhibition of cue-driven retrieval. As a result, varied sensory information that has similarities with the traumatic situation has the ability to trigger intrusive trauma memories that appear to happen in the present (10, 11). Further work concluded that integrating information relevant to the worst moments of the trauma, which may not have been available to or accessed by the individual at the time, into the memory was central to effective memory updating and to change the highly personal threatening meanings of the traumatic moment (54). For example, for a client with PTSD who during a physical assault thought they were going to die and their family would not be provided for, updating the relevant moment in memory with information only apparent after the trauma such as “I did not die, I am still providing for my family” subsequently reduced the threatening meaning and distress, and led to a reduction in intrusive trauma memories (55). With grief-related memories, updating focuses on the personal meaning of the death for the client and also the meaning of the cherished person to them. Here a memory of seeing the dying person on the day before their death may be reexperienced along with meanings such as “they are suffering unbearable pain and feel let down,” creating a sense that the death is happening again or has just happened. This feeling of “nowness” of the memories maintains a sense that the person is still suffering and contradicts the permanence of the death. Here aspects of the death that bring comfort and contradict the meanings such as “He did die but he was not alone, a nurse was with him, and he is no longer suffering” decreases distress, facilitates the acceptance that the death is in the past, and leads to a reduction in memories of seeing the person in distress [see Ehlers and Wild (56)].

Another potential indicator of the failure to process loss memories as permanent and final measured by the OG-M is the intrusive desire to both search for and comfort the deceased (57). Such intrusions are hypothesised to arise due to activation of the attachment in the absence of the deceased, which instigates a “search effort” aimed at re-establishing physical or psychological proximity (58, 59), a relationship that was supported by the strong positive correlation of the memory scales with proximity seeking behaviours. While initially the loss is not fully conceived as permanent resulting in the searching described here, Horowitz (60) and others have hypothesised that only through emotional tolerance of repeated attempts at reunion does a revised understanding of the loss as irrevocable develop. Clinical approaches that have evolved from this understanding have focused on facilitating an internalised psychological connection to the deceased to act as a “secure base” in the absence of physical proximity (61). Techniques such as imaginal conversations with the deceased (62) and restorative retelling in which the bereaved person imaginally comforts their dying loved one in a way that was denied to them in the actual circumstance (63) have been reported as helpful.

The OG-M and OG-M-S provide a comprehensive research and clinical tool for measuring aspects of loss memories relevant to poor loss memory integration. However, there are some limitations that should be considered. The study used online self-report measures to measure psychopathology making the probable diagnoses reported here less reliable than formal clinical interviews. Also previous research has suggested that self- report measures may overestimate the prevalence of mental health problems (64). Therefore, future research investigating memory characteristics in severe and enduring grief would benefit from utilising a sample diagnosed via clinical interviews to better understand the role of the OG-M in predicting psychopathology. The measurement of PGD according to ICD-11 criteria was approximated using items derived from other validated measures of PGD and PTSD, and as such we cannot fully rule out some measurement error. However, reliability of these items was good or excellent in all samples. Future research able to more closely measure ICD-11 criteria, including the item related to blame, should be conducted to ensure the generalisability of the findings. Finally, the sample employed here were largely female and Caucasian. With recent research demonstrating a wide variation of PGD prevalence across cultures (65, 66) it will be important to replicate this work in more diverse populations to ensure the cultural relevance of loss memory characteristics.

Despite these limitations, this study presents the comprehensive OG-M and shortened OG-M-S questionnaires for the assessment of loss-related memory characteristics after bereavement and demonstrates their capacity to serve as metrics for poor loss memory integration relevant to the development and maintenance of PGD and importantly, its treatment.

## Data Availability Statement

The datasets presented in this article are not readily available because no data arising from this study can be shared publicly in line with the terms of the ethics agreement provided by the University of Oxford Medical Sciences Inter-Divisional Research Ethics Committee (MS-IDREC-C1-2015-230 and MS-IDREC-C1-2015-231), which stipulated that no data could be shared with anyone outside of the research team. The relevant ethics board can be contacted on ethics@medsci.ox.ac.uk Readers can request analyses scripts and corresponding outputs from the Centre for Anxiety Disorders and Trauma at the University of Oxford, The Old Rectory, Paradise Square, Oxford, OX1 1TW. Email: oxcadat.enquiries@psy.ox.ac.uk.

## Ethics Statement

The studies involving human participants were reviewed and approved by the University of Oxford Medical Sciences Inter-Divisional Research Ethics Committee (MS-IDREC-C1-2015-230 and MS-IDREC-C1-2015-231). The patients/participants provided their written informed consent to participate in this study.

## Author Contributions

KS and AE developed the study concept and design. KS performed the data collection and statistical analysis under the supervision of AE. KS drafted the manuscript. AE and JW provided the critical revisions. All authors contributed to the questionnaire development.

## Author Disclaimer

The views expressed are those of the author(s) and not necessarily those of the NHS, the NIHR, or the Department of Health. These funding sources did not have a role in the design of the study, collection, analysis, and interpretation of the data and in writing the manuscript.

## Conflict of Interest

The authors declare that the research was conducted in the absence of any commercial or financial relationships that could be construed as a potential conflict of interest.

## Publisher’s Note

All claims expressed in this article are solely those of the authors and do not necessarily represent those of their affiliated organizations, or those of the publisher, the editors and the reviewers. Any product that may be evaluated in this article, or claim that may be made by its manufacturer, is not guaranteed or endorsed by the publisher.

## Footnotes

1 See Supplementary Material for EFA and CFA factor loadings of the OG-M-S.

2 See Supplementary Material for results of cross-lagged analyses of short scale.
