# Supplementary material for: Psychometric Characteristics of the Oxford Grief Memory Characteristics Scale and Its Relationship With Symptoms of ICD-11 and DSM-5-TR Prolonged Grief Disorder
Source: Front Psychiatry. 2022 Mar 16;13:814171. doi: 10.3389/fpsyt.2022.814171 (PMC8970310; doi:10.3389/fpsyt.2022.814171)
Supplement: Supplementary file 1 [file Data_Sheet_1.PDF]

Supplementary material

Table A1.

Means and standard deviations of memory scale items and global scores across time points in the longitudinal sample

	Loss-related memory characteristics items – Shortened Scale and Removed Items	T1 M (SD)	T2 M (SD)	T3 M (SD)
1	In the last week, approximately how often did unwanted memories of the loss pop into your mind?	2.50 (1.27)	1.94 (1.22)	1.54 (1.25)
2	How distressing were these memories?	2.36 (1.23)	1.99 (1.18)	1.67 (1.24)
3	To what extent were you reliving your experience of the loss?	2.37 (1.37)	1.79 (1.34)	1.42 (1.32)
4	The physical pain of loss is something I carry everywhere.	2.42 (1.44)	1.75 (1.45)	1.65 (1.47)
5	When I remember the loss it feels unreal.	2.63 (1.30)	2.04 (1.42)	1.88 (1.40)
6	Seeing other people with their partners or families makes me painfully aware of my loss.	2.31 (1.50)	2.09 (1.46)	1.86 (1.45)
7	Many different things trigger distressing memories of the loss.	2.44 (1.35)	2.12 (1.29)	1.68 (1.32)
8	If my mind is distracted from my grief for a while it will hit me like a wave later.	2.21 (1.44)	1.75 (1.44)	1.29 (1.41)
9	When I remember the loss, I feel the same emotions I felt at the time.	2.40 (1.26)	1.87 (1.26)	1.68 (1.29)
10	When I try to remember good times we have shared, memories of the loss pop up.	2.34 (1.31)	1.97 (1.26)	1.68 (1.33)
11	The loss feels as if part of my body is missing.	1.90 (1.60)	1.54 (1.56)	1.35 (1.54)
12	Many things in everyday life trigger overwhelming sadness.	2.39 (1.35)	1.81 (1.37)	1.51 (1.29)
13	Even nice memories cause me to feel significant pain.	2.01 (1.32)	1.62 (1.30)	1.36 (1.23)
14	When I remember something I did in the past; it feels like I am no longer the same person.	1.80 (1.47)	1.81 (1.43)	1.77 (1.44)
15	I feel a strong urge to comfort [-].	2.27 (1.54)	1.90 (1.54)	1.76 (1.51)
16	I struggle to remember positive times without [-].	1.15 (1.38)	0.98 (1.22)	0.83 (1.17)
R	When I think about [-] I will always think about how they died.	2.15 (1.40)	1.91 (1.34)	1.74 (1.28)
R	To what extent did they seem to be happening now instead of being something from the past?	1.70 (1.34)	1.14 (1.20)	.95 (1.17)
R	The memories of [-]'s death make my body ache with overwhelming fatigue.	1.95 (1.54)	1.52 (1.48)	1.27 (1.42)
R	I am reminded of the loss for no apparent reason.	2.74 (1.43)	2.36 (1.28)	2.00 (1.29)
R	When I think of [-] all I can remember is their suffering.	1.36 (1.27)	1.13 (1.15)	1.05 (1.14)
R	My memories of [-] are so vivid it feels like they are here.	1.96 (1.40)	1.63 (1.33)	1.30 (1.32)
R	When I remember things we did together it feels like I am no longer the same person.	1.86 (1.41)	1.83 (1.41)	1.66 (1.45)
R	Memories of things we did together are painful.	1.56 (1.35)	1.21 (1.24)	1.10 (1.22)
R	Looking at a calendar mainly reminds me of the bad things that happened on those days.	1.40 (1.40)	1.37 (1.44)	1.36 (1.36)
R	I hardly remember anything that I did without [-].	.89 (1.33)	.93 (1.35)	.83 (1.22)
R	I find myself suddenly overcome to find [-].	1.59 (1.53)	1.16 (1.37)	1.03 (1.32)
	Global mean OG-M (SD) range 0-108	54.20 (25.43)	44.07 (26.40)	38.65 (27.02)
	Global mean OG-M-S (SD) range 0 - 64	35.19 (16.01)	28.25 (16.78)	24.50 (17.13)

Table A2.

Analyses of the Oxford Grief loss-related memory characteristics short scale (OG-M-S)

		Factor	
		1	
	Loss-related memories items	EFA	CFA
1	In the last week, approximately how often did unwanted memories of the loss pop into your mind?	.69	.77
2	How distressing were these memories?	.85	.85
3	To what extent were you reliving your experience of the loss?	.86	.82
4	The physical pain of loss is something I carry everywhere.	.86	.82
5	When I remember the loss it feels unreal.	.69	.61
6	Seeing other people with their partners or families makes me painfully aware of my loss.	.67	.66
7	Many different things trigger distressing memories of the loss.	.84	.85
8	If my mind is distracted from my grief for a while it will hit me like a wave later.	.82	.81
9	When I remember the loss, I feel the same emotions I felt at the time.	.61	.68
10	When I try to remember good times we have shared, memories of the loss pop up.	.69	.74
11	The loss feels as if part of my body is missing.	.80	.79
12	Many things in everyday life trigger overwhelming sadness.	.84	.82
13	Even nice memories cause me to feel significant pain.	.83	.76
14	When I remember something I did in the past; it feels like I am no longer the same person.	.70	.65
15	I feel a strong urge to comfort [-].	.58	.68
16	I struggle to remember positive times without [-].	.66	.64

Notes: EFA (N = 348) CFA (N=328). Factor labelled as Loss-Related Memory Characteristics. All factor loadings significant to $p < .05$.

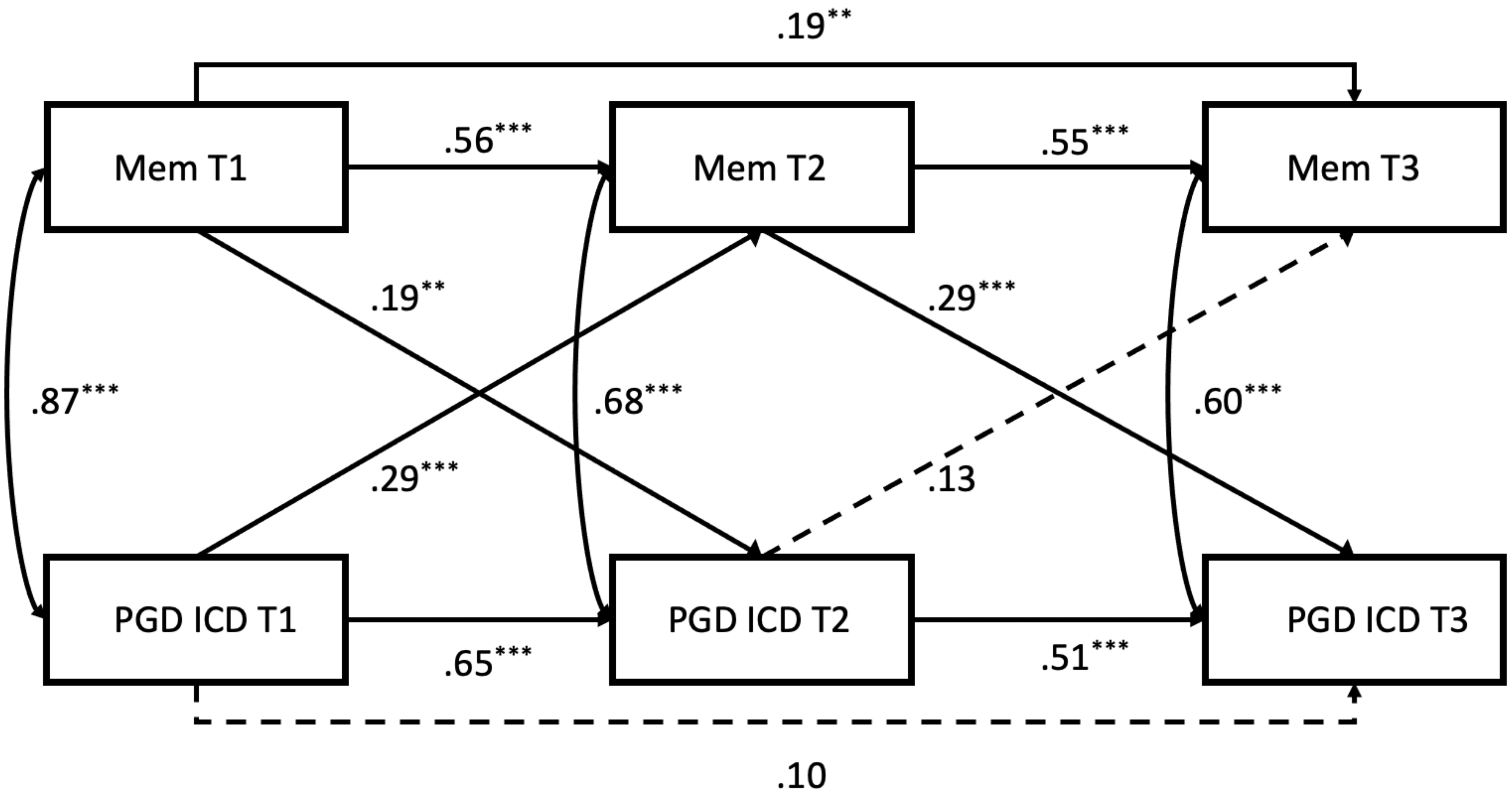

Fig A1. Autoregressive cross-lagged model of PGD ICD-11 and loss-related memory characteristics (short scale).
Note: Standardised coefficients are shown. Broken lines indicate non-significant paths. MEM = The Oxford Grief Memory Characteristics – Short Scale (OG-M-S) PGD ICD = Prolonged Grief Disorder according to the ICD-11 diagnostic criteria.
Asterisks indicate significant associations ($^{*}p < .05$, $^{**}p < .01$, $^{***}p < .001$).
$\chi^2 = 1.64$, df = 2, $p > .05$, RMSEA = .00 (.00 - .11), CFI = 1.00 TLI = 1.00

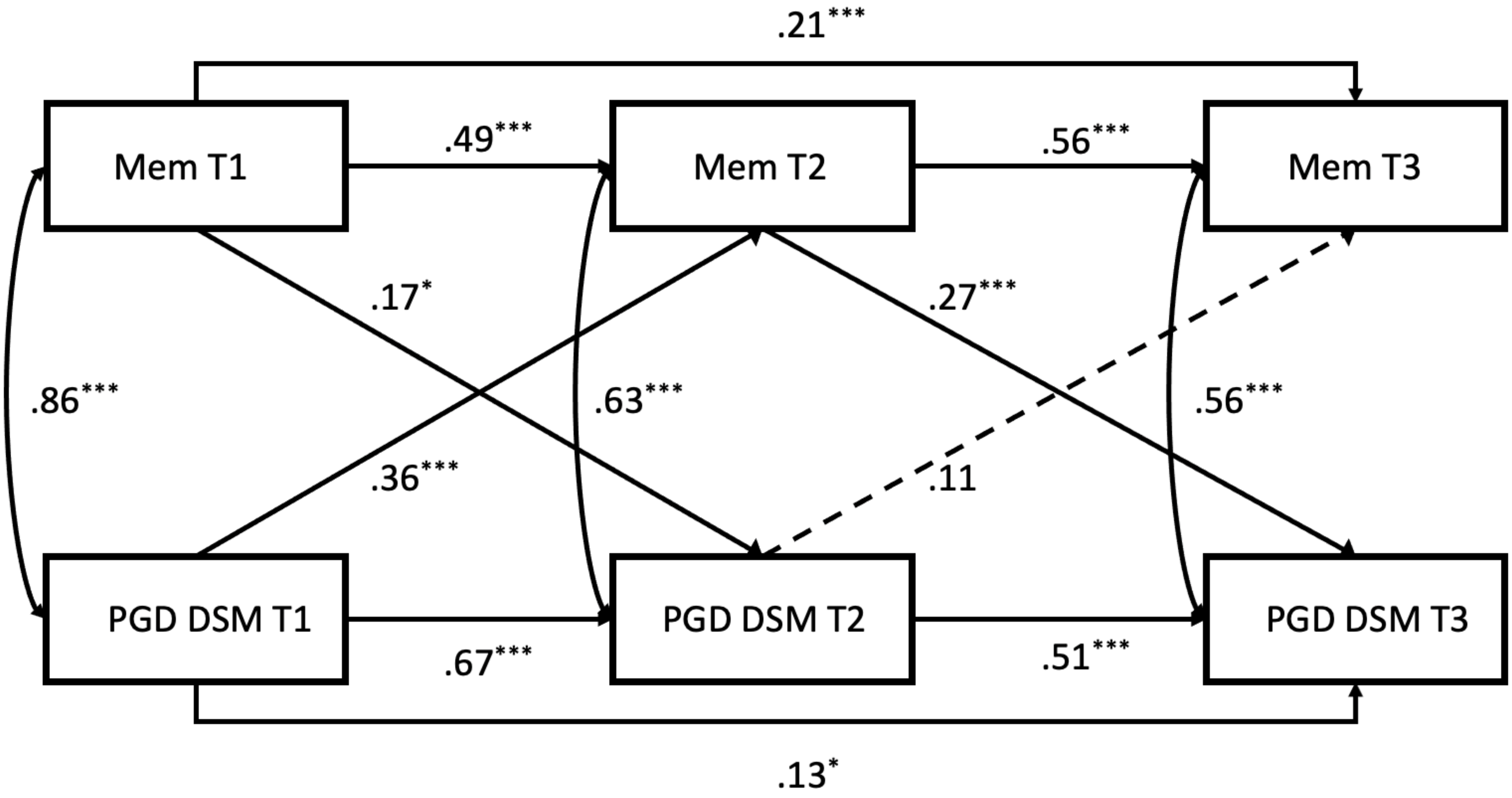

Fig A2. Autoregressive cross-lagged model of PGD DSM-5-TR and loss-related memory characteristics (short scale).
Note: Standardised coefficients are shown. Broken lines indicate non-significant paths. MEM = The Oxford Grief Memory Characteristics – Short Scale (OG-M-S) PGD DSM= Prolonged Grief Disorder according to the DSM-5-TR diagnostic criteria.
Asterisks indicate significant associations ($^{*}p < .05$, $^{**}p < .01$, $^{***}p < .001$).
$\chi^2 = 2.28$, df = 2, $p > .05$, RMSEA = .039 (.00 - .13), CFI = 1.00, TLI = 1.00
